# Cancer: Strong Signal for Cell Phone Effects

**DOI:** 10.1289/ehp.116-a422

**Published:** 2008-10

**Authors:** M. Nathaniel Mead

With 3 billion cell phone users worldwide and more than 260 million in the United States alone—among them 46% of U.S. children aged 8–12, according to Nielsen Mobile figures released 10 September 2008—human exposure to low-energy radiation in the 800- to 2,000-megahertz range is at an all-time high. The most recent attempt to systematically review the epidemiologic evidence for increased risk of brain tumors related to cell phone use indicates that repercussions from this global experiment are coming to light. In a meta-analysis published in the May 2008 issue of the *International Journal of Oncology*, Swedish researchers found significant associations between long-term cell phone use and brain tumor risk.

“We found that cell phone use is linked to gliomas [malignant brain tumors] and acoustic neuromas [benign tumors of the brain’s auditory nerve] and are showing up after only ten years,” says lead author Lennart Hardell, an oncologist and cancer epidemiologist at University Hospital in Örebro, Sweden. Specifically, for studies that included at least 10 years of exposure, there was a doubling in the risk of gliomas for ipsilateral (same-side) but not contralateral (opposite-side) exposures to the head (as reflected by which hand the subject typically used to hold his/her cell phone). A 2.4-fold increase in risk was seen for acoustic neuromas due to ipsilateral exposures, whereas no increased risk occurred for meningiomas (tumors that occur in the membranes covering the brain and spinal cord).

“Clearly we need more studies of long-term cell phone usage to better assess the cancer risks,” says coauthor Michael Carlberg. Cell phones have been in mainstream usage for only a decade or so, and yet radiation-induced brain tumors normally take about 10–15 years to develop, according to the American Cancer Society.

Hardell’s research team was itself the source of several studies included in the meta-analysis. In the October 2006 issue of the *World Journal of Surgical Oncology*, the investigators reported a 70% increased risk of grade III–IV astrocytomas (highly aggressive brain tumors) for analog cell phone users. This same study found a nearly 4-fold increase in risk for acoustic neuromas after 15 years of exposure to analog cell phones. Notably, there was no increased risk for testicular cancer, B-cell lymphoma, or salivary gland tumors, suggesting that the findings were not due to observational or recall bias, as such bias should have existed for all tumor types.

To address whether their earlier studies may have skewed the conclusions of their 2008 meta-analysis, the team omitted their own studies from the analysis and still found significantly increased risk for gliomas and nonsignificantly increased risk for acoustic neuromas (50% and 210% increases, respectively) for ipsilateral exposures. “We are now seeing a consistent pattern of increased risk for glioma and acoustic neuroma,” says coauthor Kjell Hansson Mild, a radiation physicist at Umeå University, Sweden. “Not only our own studies are showing this but also all other studies that have included at least ten years as a latency period.”

Emerging evidence suggests that children may be more vulnerable to the potential carcinogenic effects of cell phones and other microwave-emitting technologies. “Concerns about children’s potential vulnerability to RF [radiofrequency] fields have been raised because of the potentially greater susceptibility of their developing nervous systems,” says Leeka Kheifets, an epidemiology professor at the University of California, Los Angeles, and former director of the Electric Power Research Institute EMF research program. “In addition, their brain tissue is more conductive, RF penetration is greater relative to head size, and they will have a longer lifetime of exposure [although the degree of risk for any carcinogen will be primarily determined by the exact timing and magnitude of exposure].”

The importance of a thinner skull and differing dielectric properties is confirmed by a study in the 7 June 2008 issue of *Physics in Medicine and Biology* showing that a child’s brain absorbs up to twice as much RF as an adult brain. Children today will experience a longer period of exposure because they start using cell phones at an earlier age, according to Hardell; this might be important, because cumulative dose seems to have a strong influence on increased risk of brain tumors. Kheifets adds, however, that “data are lacking on effects of exposures on brain tumors in children . . . [and] other health effects need to be looked at as well.”

The wireless industry takes a cautious view of the research. “The weight of the scientific evidence and the conclusions of a large number of expert scientific reviews show that wireless phones do not pose a health risk,” says Joseph Farren, assistant vice president for public affairs with CTIA–The Wireless Association. “The industry supports continued research as technology continues to evolve, but wishes to stress the fact that there is a consensus among leading health organizations regarding published scientific research showing no reason for concern.”

Hardell concedes it is too soon to determine a safe limit for cell phone use. “Can we say that a ten-minute call is equal to ten one-minute calls?” he asks. “Until we answer such questions, we cannot establish a new limit or even state which parameters or units help define that limit. Nonetheless, since we do see an increased risk of brain tumors, it is necessary to apply the precautionary principle in this situation, especially for long-range exposures that are likely to affect children in particular.” In practice, this might involve limiting children’s use of cell phones and using speaker phones to minimize direct exposure to the head.

## Figures and Tables

**Figure:**
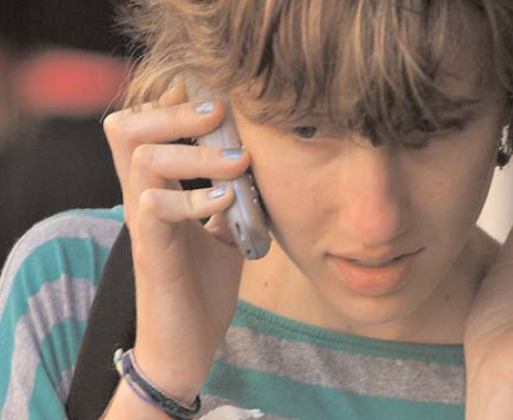
In July 2008 market research firm MultiMedia Intelligence reported that more than 16 million U.S. teens use cell phones.

